# Prognostic value of late enhancement in cardiac magnetic resonance in patients with dilated cardiomyopathy: a meta-analysis

**DOI:** 10.1186/1532-429X-18-S1-P114

**Published:** 2016-01-27

**Authors:** Francesco Secchi, Marcello Petrini, Paola M Cannao, Marco Alì, Giovanni Di Leo, Massimo Lombardi, Francesco Sardanelli

**Affiliations:** Radiology, IRCCS Policlinico San Donato, Milan, Italy

## Background

To systematically review the prognostic value of late gadolinium enhancement (LGE) at cardiac magnetic resonance (CMR) in patients with dilated cardiomyopathy (DCM).

## Methods

A literature search was performed on Medline and Embase for original articles estimating the LGE prognostic value in patients with DCM. Original articles had to assess mortality for cardiac and non-cardiac causes, sudden cardiac death, sudden death avoided, and hospitalization for cardiac failure. Heterogeneity (I^2^) was evaluated using the Cochrane Q statistics: P-value <0.100 were considered significant. Pooled odd ratio (OR) and 95% confidence interval (CI: 95%) were calculated using Comprehensive Meta-Analysis.

## Results

Out of 691 articles initially retrieved, 6 prospective clinical trials were selected for a total of 1,017 patients. All analyzed studies were performed using a 1.5-T MR unit. LGE was positively correlated with all considered clinical outcomes. Pooled mortality for all causes showed I^2^=33% p = 0.202) and OR=2.6 (95%CI 1.7-4.0; *p*<0.001); hospitalization for cardiac failure showed I^2^=24% (*p*=0.257) and OR=2.7 (95%CI 1.8-4.1; *p*<0.001); sudden cardiac death showed I^2^=0% (*p*=0.895) and OR=3.2 (95%CI 1.6-6.3; *p*=0.001); death for cardiac causes showed I^2^=0% (*p*=0.782) and OR=3.5 (95%CI 2.2-5.7; *p*<0.001); sudden death avoided showed I^2^=0% (*p*=0.815) and OR=6.3 (95%CI 3.4-11.6; *p*<0.001).

## Conclusions

LGE at CMR in patients with CMD is closely related to a more negative prognosis if compare to patients without LGE.

